# Fulminant Primary Biliary Cholangitis-Autoimmune Hepatitis (PBC-AIH) Overlap Syndrome in a 27-Year-Old Woman With Childhood-Onset AIH: Steroid-Refractory Decompensation Necessitating Urgent Transplant Evaluation

**DOI:** 10.7759/cureus.87519

**Published:** 2025-07-08

**Authors:** Ebram Said, Yordanos Tafesse, Hossam R Elbenawi, Shyam Chalise, George Atia

**Affiliations:** 1 Internal Medicine, Ascension Saint Joseph Hospital, Chicago, USA; 2 Internal Medicine, Mansoura University School of Medicine, Mansoura, EGY; 3 Gastroenterology and Hepatology, Ascension Health, Chicago, USA

**Keywords:** autoimmune hepatitis (aih), autoimunne hepatites-primary biliary cholangitis overlap, hepatic failure, hepatocellular liver injury, primary biliary cholangitis (pbc)

## Abstract

Autoimmune hepatitis (AIH) and primary biliary cholangitis (PBC) represent distinct autoimmune liver diseases, each with characteristic clinical, serological, and histological features. Rarely, patients may exhibit an overlap syndrome, presenting diagnostic and therapeutic challenges. We describe a 27-year-old woman with a longstanding history of childhood-onset AIH who subsequently developed clinical and serologic features consistent with PBC, fulfilling the Paris criteria for AIH-PBC overlap syndrome. Despite aggressive treatment with high-dose corticosteroids, she experienced rapid clinical deterioration, developing acute hepatic encephalopathy and requiring intensive care unit admission. A liver biopsy confirmed the coexistence of interface hepatitis (typical of AIH) and florid bile duct lesions (characteristic of PBC). Notably, the patient had a steroid-refractory course, systemic autoimmune comorbidities including ulcerative colitis and pyoderma gangrenosum, and exhibited atypical early-onset disease progression. Ultimately, due to severe and refractory liver failure, she required urgent liver transplantation evaluation. This case highlights the aggressive clinical course and critical management complexities of AIH-PBC overlap syndrome, emphasizing the importance of early recognition, comprehensive histologic evaluation, combined immunosuppressive and cholestatic therapies, and expedited referral for transplantation in refractory cases.

## Introduction

Autoimmune hepatitis (AIH) and primary biliary cholangitis (PBC) represent distinct autoimmune liver disorders characterized by hepatocellular inflammation and cholestatic bile duct injury, respectively [1]. Occasionally, patients present with overlapping serologic, biochemical, and histologic features of both conditions, fulfilling the criteria for an overlap syndrome [2]. Clinically, these patients often exhibit nonspecific symptoms such as fatigue, arthralgia, and pruritus, accompanied by elevations in both transaminases and cholestatic enzymes [2,3]. In the absence of universally accepted diagnostic standards, the Paris criteria, requiring two of three defining features for each disease, are most commonly applied, although evolving clinical presentations may escape this framework [4]. Given the potential for rapid fibrosis progression or liver failure, prompt and accurate recognition is critical, since combined immunosuppressive therapy and ursodeoxycholic acid (UDCA) are often necessary [2]. We describe a young woman with longstanding AIH who developed fulminant AIH-PBC overlap syndrome, highlighting critical diagnostic and therapeutic challenges.

## Case presentation

A 27-year-old woman with a history of ulcerative colitis, hypothyroidism status post total thyroidectomy for papillary thyroid carcinoma (five years prior), bipolar disorder, and biopsy-confirmed AIH diagnosed at age 12, presented with six weeks of progressive fatigue, jaundice, and generalized skin eruptions following recent travel to Mexico. She was urgently referred to the emergency department by her gastroenterologist after the development of new-onset abdominal pain, and abnormal liver function tests were identified during the evaluation of her worsening symptoms. Her AIH had previously been well-controlled and in sustained remission on immunosuppressive therapy with corticosteroids, which had been discontinued several years prior due to long-term disease stability. She was also receiving oral mesalamine for ulcerative colitis.

Initial laboratory investigations revealed a mixed hepatocellular and cholestatic pattern of liver injury. Notable abnormalities included elevated aspartate aminotransferase (AST), alanine aminotransferase (ALT), total bilirubin, alkaline phosphatase (ALP), gamma-glutamyl transferase (GGT), international normalized ratio (INR), and prothrombin time (PT) values. The patient reported abdominal pain and generalized fatigue and was noted to have diffuse skin eruptions. Further serologic workup demonstrated a positive antinuclear antibody (ANA) with a speckled pattern, positive antismooth muscle antibody (ASMA), positive F-actin-specific immunoglobulin G (IgG), and positive antimitochondrial M2 antibody (AMA-M2). Total serum IgG levels were also elevated. A complete summary of initial laboratory parameters, reference ranges, and interpretive status is provided in Table 1.

**Table 1:** Summary of laboratory investigation results ELISA: enzyme-linked immunosorbent assay

_Parameter_	_Obtained value and status_	_Reference range/interpretation_
Aspartate aminotransferase (AST)	302 U/L (elevated)	13-39 U/L
Alanine aminotransferase (ALT)	519 U/L (elevated)	7-52 U/L
Alkaline phosphatase (ALP)	311 U/L (elevated)	40-129 U/L
Gamma-glutamyl transferase (GGT)	107 U/L (elevated)	9-48 U/L
Total bilirubin	7.6 mg/dL (elevated)	0.1-1.2 mg/dL
Prothrombin time (PT)	24.4 sec (elevated)	10.1-13.1 sec
International normalized ratio (INR)	2.3 (elevated)	0.9-1.1
Antinuclear antibody (ANA)-titer & pattern	Positive, 1:160, speckled	<1:80: negative; ≥1:160: positive
Total immunoglobulin G (IgG)	1,866 mg/dL (elevated)	700-1,600 mg/dL
Antismooth muscle antibody (SMA) titer	1:80 (positive)	≥1:40: positive
F-Actin IgG (smooth muscle antibody) by ELISA	>100 units (positive)	≥31: positive
Antimitochondrial antibody M2 (AMA-M2)	36.3 units (positive)	≥25: positive

The right upper quadrant ultrasound showed heterogeneous liver parenchyma with a nodular contour suggestive of cirrhosis and moderate to large-volume ascites. Magnetic resonance imaging was suggestive of cirrhotic liver morphology with signs of portal hypertension, splenomegaly, ascites, and a 5 mm cystic lesion in the pancreatic head. The incidental pancreatic cyst was not evaluated due to acute clinical priorities. A transjugular liver biopsy was performed.

Given the clinical and laboratory findings concerning the AIH flare-up, the patient was started on intravenous methylprednisolone 30 mg twice daily while awaiting biopsy results. Azathioprine (AZA) was planned pending thiopurine methyltransferase (TPMT) testing. Despite corticosteroid therapy, there was no significant improvement in liver enzymes, and the patient’s overall clinical condition worsened.

**Figure 1:**
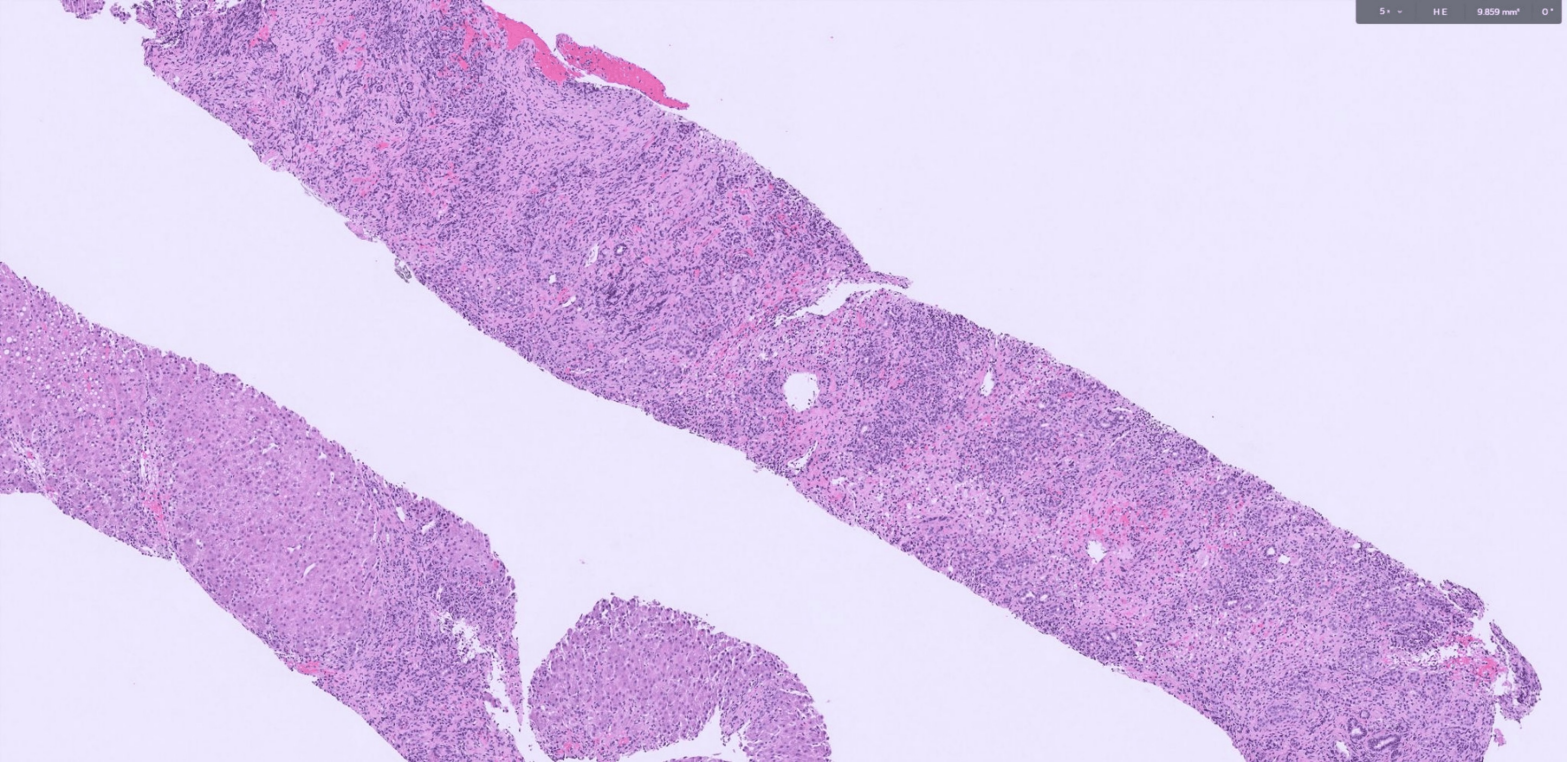
Hematoxylin and eosin (H&E)-stained liver core biopsy at low magnification Hematoxylin and eosin (H&E)-stained liver core biopsy at low magnification showing portal and periportal inflammatory infiltrates with interface hepatitis. Inflammatory cells extend from the portal tracts into surrounding hepatocytes

**Figure 2:**
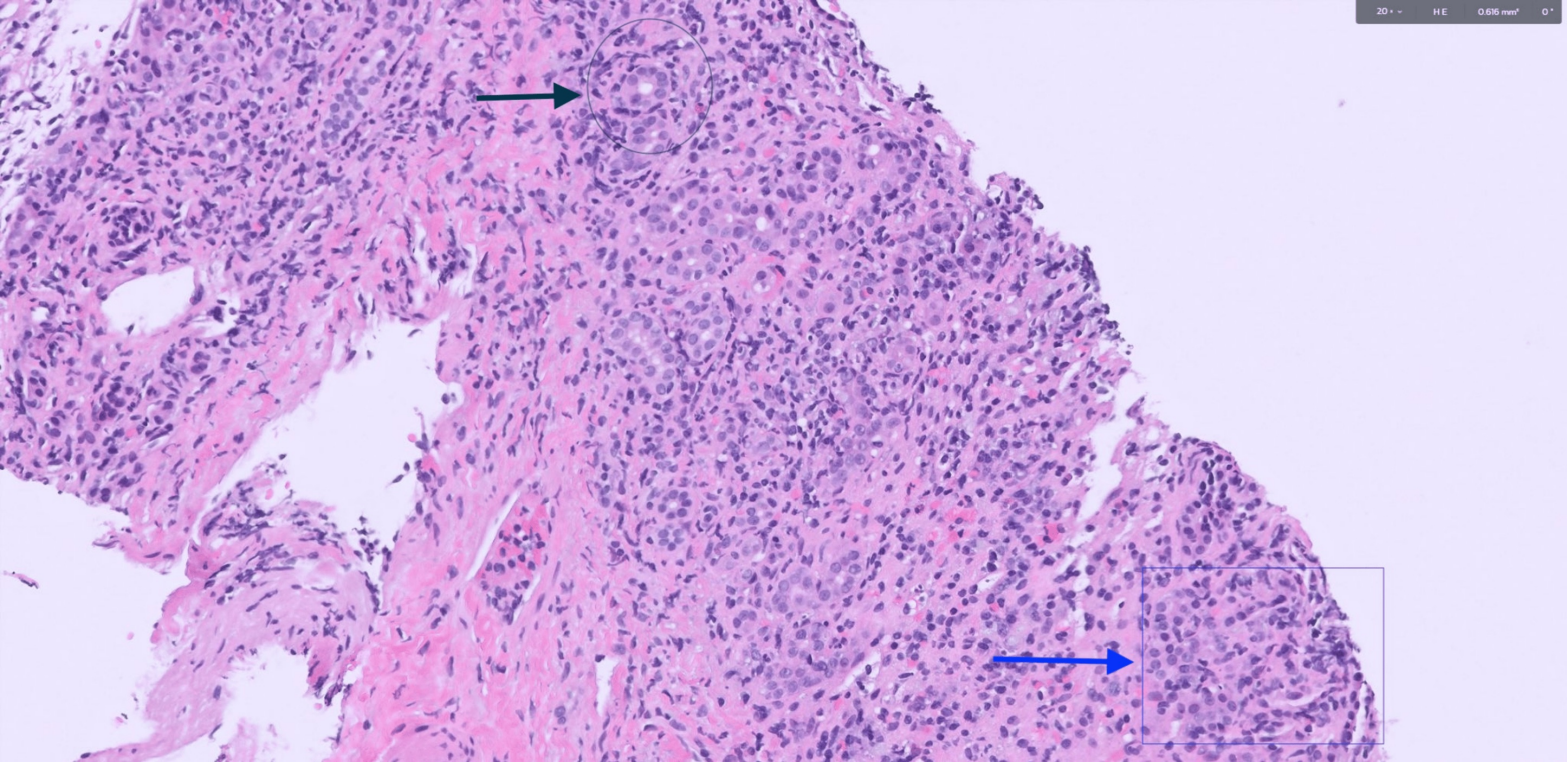
High-power hematoxylin and eosin (H&E)-stained liver biopsy AIH: autoimmune hepatitis; PBC: primary biliary cholangitis; H&E: high-power hematoxylin and eosin H&E-stained section of liver biopsy. The upper arrow highlights interface hepatitis, showing lymphoplasmacytic infiltration extending beyond the limiting plate into periportal hepatocytes. The lower arrow points to a bile duct with surrounding lymphocytic infiltrate, consistent with bile duct injury. These features are characteristic of AIH with overlapping features of AIH-PBC overlap syndrome

**Figure 3:**
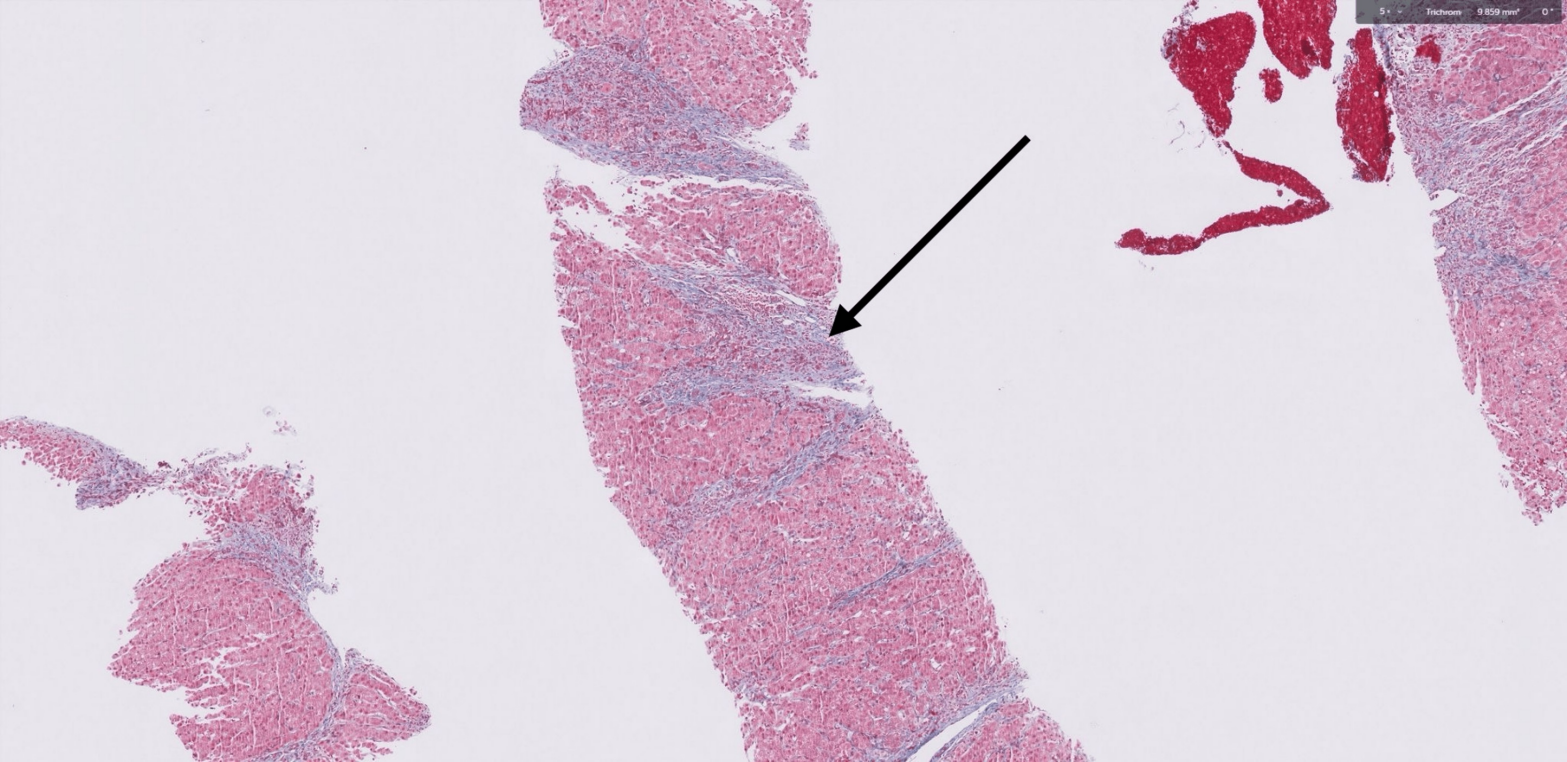
Trichrome-stained liver biopsy Trichrome-stained liver biopsy demonstrating bridging fibrosis (arrow), evidenced by blue-stained collagen extending between portal areas, consistent with stage 2 fibrosis

Liver biopsy demonstrated characteristic findings of both PBC (florid duct lesions with prominent ductular reaction) and AIH (interface hepatitis with dense lymphoplasmacytic infiltrates breaching the limiting plate) (Figures 1-2). Additionally, bridging and septal fibrosis were present (Figure 3), reflecting chronic biliary and hepatocellular injury. Based on her clinical presentation and laboratory findings, the patient met the diagnostic criteria for PBC-AIH overlap syndrome according to the Paris criteria. She had elevated ALP, positive AMA, and bile duct fibrosis consistent with PBC, along with ALT greater than five times the upper limit of normal and positive ASMA, supporting an AIH component. The presence of both hepatocellular and cholestatic injury patterns helped explain the patient’s rapid clinical deterioration and steroid-refractory disease course.

Her hospital course was complicated by the development of hepatic encephalopathy and hemodynamic instability with elevated lactate levels, necessitating transfer to the intensive care unit. She continued to clinically decompensate, with worsening hepatic function and altered mental status. Due to concern for impending septic and/or hypovolemic shock, she was initiated on antibiotics, lactulose, rifaximin, and aggressive fluid resuscitation. Given the severity of her liver failure and multiple complications, the patient was transferred to a tertiary liver transplant center, where she was listed and is awaiting liver transplantation.

## Discussion

Autoimmune liver diseases represent a complex spectrum of conditions, AIH and PBC being two distinct entities with overlapping features [5]. Overlap syndrome is a term used to represent forms of AIH that present with either AIH and PBC or AIH and primary sclerosing cholangitis. PBC-AIH overlap syndrome is a rare clinical condition wherein patients exhibit diagnostic characteristics of both diseases [6]. This case highlights an uncommon presentation of overlap syndrome in a young woman with a complex autoimmune background and rapid clinical deterioration.

PBC and AIH have traditionally been viewed as separate diseases [2]. PBC involves immune-mediated destruction of intrahepatic bile ducts, while AIH targets hepatocytes, causing interface hepatitis [3]. However, overlap syndrome blurs these boundaries. Like the case described, patients can present with fatigue and abdominal pain; additionally, they can present with pruritus [3]. The most commonly used diagnostic framework, the Paris criteria, requires patients to fulfill at least two of three criteria for each condition [2,3].

Our patient met multiple diagnostic criteria for PBC an elevated ALP at 311 U/L, positive AMA-M2, and liver biopsy showing florid bile duct lesions with bridging fibrosis. Simultaneously, she met the AIH criteria: ALT >5× upper limit of normal (519 U/L), positive ANA and ASMA, and elevated immunoglobulin G (IgG). The presence of both serologic markers and histopathologic findings from both disease spectra confirmed the diagnosis of PBC-AIH overlap syndrome.

This case is notable for several reasons. First, the patient’s young age is atypical for PBC, which more commonly affects women in their 50s [7,8]. Second, she had a longstanding history of AIH diagnosed in childhood and managed intermittently with steroids and AZA. The subsequent development of PBC features, including AMA positivity and bile duct injury on biopsy, suggests either transformation of disease or coexistence of two autoimmune hepatic processes.

Furthermore, she had other coexisting autoimmune disorders, ulcerative colitis indicating a systemic autoimmune predisposition. Another notable feature was the presence of diffuse skin eruptions, which were later attributed to pyoderma gangrenosum (PG). PG is a rare, neutrophilic dermatosis frequently associated with systemic inflammatory and autoimmune disorders such as inflammatory bowel disease (IBD) and AIH [9,10]. Its presence in this patient, who also had ulcerative colitis and multiple autoimmune conditions, further highlights the systemic autoimmune dysregulation underlying her disease. This clustering of autoimmune diseases is recognized but still poorly understood in the pathogenesis of hepatic autoimmune overlap syndromes.

Another striking aspect is the lack of response to corticosteroids, a mainstay of AIH therapy. This suggests a stronger cholestatic (PBC) component, which does not typically respond to immunosuppression alone and may require ursodeoxycholic acid (UDCA) for effective disease control [2]. The rapid decompensation despite timely initiation of corticosteroids underlines the severity of this overlap presentation and supports the notion that overlap syndromes can behave more aggressively than either condition alone. The patient's rapid decline underscored the need for urgent liver transplantation evaluation, which ultimately prompted transfer to a tertiary liver center. In advanced stages of the disease, liver transplantation is the preferred treatment option [11].

This case exemplifies the diagnostic and therapeutic complexity of overlap syndrome. A high index of suspicion is required, particularly in patients with atypical presentations, poor response to standard therapy, or features of both cholestasis and hepatocellular injury. Liver biopsy remains an essential tool in confirming the diagnosis, especially when serologies are ambiguous or when prior autoimmune liver disease evolves into a new clinical pattern.

Overlap syndrome has been associated with worse outcomes than isolated PBC or AIH [2]. Patients may progress more rapidly to cirrhosis and liver failure if not promptly and adequately treated [2]. Treatment typically involves a combination of immunosuppressive therapy either budesonide with AZA or predniso(lo)ne with AZA to address the AIH component, alongside UDCA to manage the cholestatic features of PBC. AZA is generally introduced after confirming normal TPMT activity [2,8]. In female patients of reproductive age, pregnancy should be excluded before initiating therapy, as AZA is safe for use during pregnancy, whereas mycophenolate mofetil (MMF), a second-line agent, is contraindicated [3]. In refractory or fulminant cases, early referral for liver transplantation is warranted.

## Conclusions

This case demonstrates fulminant hepatic decompensation in a young woman with childhood-onset AIH who developed steroid-refractory AIH-PBC overlap syndrome, necessitating urgent transplant evaluation. Key findings included atypical early PBC emergence despite prior AIH remission, histopathologic evidence of both interface hepatitis and florid duct lesions fulfilling the Paris criteria, systemic autoimmune comorbidities (ulcerative colitis, pyoderma gangrenosum), and rapid deterioration unresponsive to corticosteroid therapy. The definitive outcome, transfer to a transplant center, underscores the syndrome’s aggressive course, aligning with evidence of poorer prognoses than isolated autoimmune liver diseases. This underscores the imperative for early liver biopsy, combined immunosuppressive/UDCA therapy, and expedited transplant assessment in refractory or atypical presentations.
